# Terahertz Emission via Optical Rectification in a Metal-Free Perovskite Crystal

**DOI:** 10.1021/acsphotonics.3c00918

**Published:** 2023-10-18

**Authors:** Nathaniel P. Gallop, Dumitru Sirbu, David Walker, James Lloyd-Hughes, Pablo Docampo, Rebecca L. Milot

**Affiliations:** † Department of Physics, University of Warwick, Coventry CV4 7AL, U.K.; ‡ School of Mathematics, Statistics and Physics, Newcastle University, Newcastle upon Tyne NE1 7RU, U.K.; § School of Chemistry, University of Glasgow, Glasgow G12 8QQ, U.K.

**Keywords:** terahertz emission, perovskites, spectroscopy

## Abstract

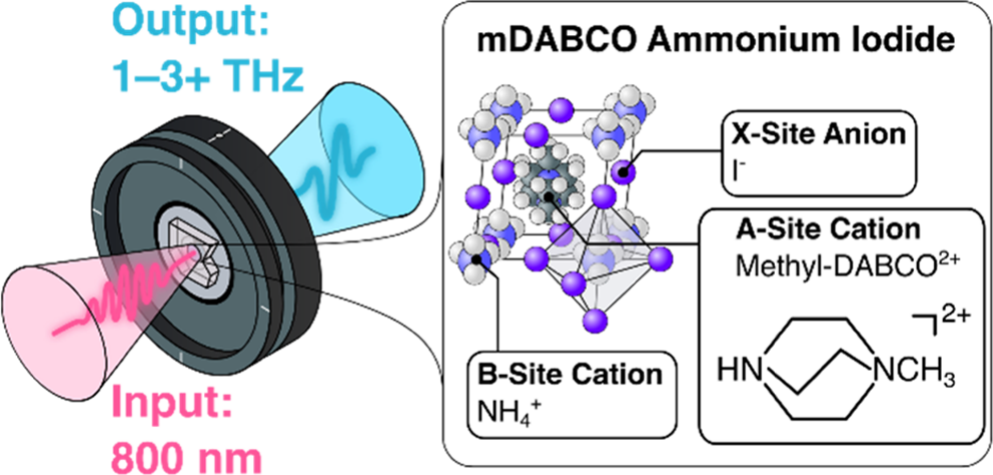

We report on the emission of high-intensity pulsed terahertz radiation from the metal-free halide perovskite single crystal methyl-DABCO ammonium iodide (MDNI) under femtosecond illumination. The power and angular dependence of the THz output implicate optical rectification of the 800 nm pump as the mechanism of THz generation. Further characterization finds that, for certain crystal orientations, the angular dependence of THz emission is modulated by phonon resonances attributable to the motion of the methyl-DABCO moiety. At maximum, the THz emission spectrum of MDNI is free from significant phonon resonances, resulting in THz pulses with a temporal width of <900 fs and a peak-to-peak electric field strength of approximately 0.8 kV cm^–1^—2 orders of magnitude higher than any other reported halide perovskite emitters. Our results point toward metal-free perovskites as a promising new class of THz emitters that brings to bear many of the advantages enjoyed by other halide perovskite materials. In particular, the broad tunability of optoelectronic properties and ease of fabrication of perovskite materials opens up the possibility of further optimizing the THz emission properties within this material class.

## Introduction

Over the past two decades, there has been an increasing amount of attention paid to the unique ways in which THz radiation interacts with materials, which has led researchers to explore the feasibility of THz technologies in a broad array of potential applications, including security,^1^ biomedicine,^2^ solid-state physics,^3−5^ chemical recognition,^6^ and high-speed wireless communications,^7^ among others. The development of these applications has motivated the continued search for brighter and more efficient sources of THz radiation and has led to a variety of novel means utilizing ultrafast lasers, including optical rectification (OR) in nonlinear crystals,^8−12^ the use of photoconductive antennas,^13^ and spintronic emitters that exploit the inverse spin-Hall effect.^14^ Of the various techniques of generating THz radiation, optical rectification is among the most efficient and is used in many applications in which high-intensity THz radiation is required.^12^ However, the strict symmetry and structural requirements of materials to be employed in OR applications limit their prevalence. The development of an inexpensive, easy-to-synthesize material platform for optical rectification would aid in stimulating the further development of optical rectification as a means of THz generation.

One potentially promising class of materials for such THz emitters are the so-called halide perovskites. Halide perovskites have attracted a great deal of industrial and academic interest as next-generation high-performance photonic and (opto)electronic materials, in particular as low-cost photovoltaic absorbers.^15^ Researchers have also identified potential applications in areas as diverse as electrocaloric refrigeration,^16^ X-ray detection, laser emission,^17^ and nonvolatile memory,^18^ among others. The diversity of potential applications of halide perovskites has been driven in no small part by their ease of fabrication and high degree of tunability of their optoelectronic properties such as bandgap absorption and charge carrier mobility. In particular—and in contrast to many other materials—halide perovskites possess explicit and easily predictable structural behaviors (for example, crystal structures via the Goldschmidt tolerance factor), meaning that protocols can quickly be adapted to change the composition of ionic species that comprise the crystal structure; this enables their rational and targeted material design, while also facilitating the optimization of existing material systems.^19−22^

However, despite the potential advantages enjoyed by halide perovskites, and despite reports demonstrating second-order optical nonlinearity in a range of halide perovskite materials (an essential criterion for the development of THz emitters that exploit optical rectification), only a small number of previous studies have investigated THz emission from prototypical halide perovskites (e.g., methylammonium lead iodide, formamidinium lead iodide) as a means to probe a variety of optoelectronic and structural processes, including spin–orbit coupling,^23,24^ electron–phonon interactions,^25^ and surface defects.^26^ In all of these examples, the intensity of the emitted radiation was extremely low (often several orders of magnitude lower than other high-performance THz emission materials). Moreover, the mechanisms of THz generation in these studies relied on the resonant absorption of an excitation beam either driving the generation of injection/shift currents,^23,24^ surge currents,^26^ or the photo-Dember effect.^25^ This need for resonant excitation has motivated the development of relatively complicated nanostructuring approaches^27^ that diminish the advantages of easy fabrication normally enjoyed by hybrid perovskites. For these reasons, it has not yet been demonstrated that halide perovskites are viable materials to exploit as THz emitters in their own right.

**Figure 1:**
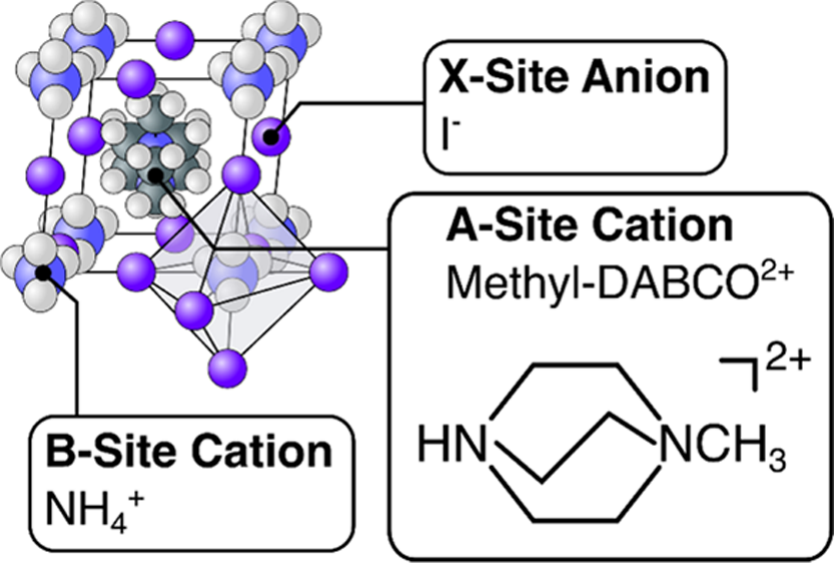
Structure of methyl-DABCO ammonium triiodide (MDNI). As with other halide perovskites, the B-site and halide species form an octahedral cage into which the A-site species are located.

To this end, we demonstrate the potential of a relatively new class of halide perovskites—termed metal-free perovskites, to find practical use as THz emitters. These materials have the potential to leverage many of the beneficial properties of hybrid perovskites—including low cost, ease-of-synthesis, and optoelectronic tunability, while also avoiding the issues of toxicity and instability that affect the more common lead- and tin-containing halide perovskites. Our metal-free perovskite of choice is *N*-methyl-1,4-diazabicyclo[2.2.2]octan-1-ium (methyl-DABCO) ammonium iodide (MDNI), a simplified structure of which is given in Figure 1. By analogy with their hybrid inorganic–organic counterparts, such as methylammonium lead triiodide (MAPbI_3_) and formamidinium lead triiodide (FAPbI_3_), metal-free perovskites comprise an organic A-site cation (here, methyl-DABCO^2+^), which occupies a cavity created by the B- and X-site ions (here, NH_4_^+^, and I^–^, respectively). Structural characterization of MDNI reveals that it crystallizes in the noncentrosymmetric *R*3 space group,^28^ while previous theoretical and experimental studies investigating the applicability of metal-free perovskites as candidates for second-harmonic generation have identified a nonzero χ^(2)^,^28−30^ factors that point toward the suitability of MDNI as a THz emitter.

In this article, we experimentally demonstrate the suitability of MDNI as a nonlinear optical medium for THz generation and assay its performance through analysis of its THz generation properties. Illumination of a (100) cut MDNI single crystal with sub-100 fs 800 nm laser pulses results in broadband THz radiation, with emission limited by the bandwidth of the electro-optical detection system. Through characterization of the power and angular dependencies of the THz emission, we implicate optical rectification as the mechanism of THz generation. We find that the emitted THz field of MDNI is significantly stronger, by 2 orders of magnitude, than those of other halide perovskites; encouragingly, we find that the THz output of MDNI is more comparable to other high-performance organic nonlinear optical materials. Given the relative nascence of the material and the need for further exploration and optimization of its properties, our results clearly demonstrate the potential of all-organic perovskite systems as efficient THz emitters.

## Experimental Section

### Synthesis of MDNI Crystals

1,4-Diazabicyclo[2.2.2]octane (DABCO), HI (57% w/w stabilized using 1.5% H_3_PO_3_), and NH_4_ were purchased and used as received. The as-received DABCO was converted to *N*-methyl-1,4-diazabicyclo[2.2.2]octan-1-ium (MDABCO) iodide using a previously described protocol.^31,32^ The production of polycrystalline MDNI from these precursors has likewise been described elsewhere.^28^

To produce the large MDNI crystals employed in this study, 0.8 g/mL solution in DIW was prepared at 90 °C and filtered hot through a hydrophilic 0.45 μm filter. Cooling the solution to RT results in submillimeter crystal seeds. Addition of one crystal seed to a fresh solution is followed by slow cooling to 50 °C at a rate of 0.5 °C/h. The crystal is further left to grow in the mother liquor at 50 °C for another 40 h. At this stage, the MDNI crystal is removed, dried under mild vacuum for 1 h, and polished with “dry” abrasive paper. After polishing, the crystal dimensions were roughly 5 mm × 7 mm × 1 mm.

To confirm the orientation of our MDNI crystal in the laboratory frame, we acquired single crystal X-ray diffraction (XRD) patterns of MDNI. To characterize the misorientation of our crystal and to establish the primary and secondary Miller indices, we compared our obtained 2θ values for the [102] and [200] peaks with their expected positions, using a previously reported structure for the *R*3 phase of MDNI (*a* = *b* = *c* = 7.259 A, α = β = γ = 84.747°).^33^ Based on this analysis, we find the misorientation of the primary and secondary Miller indices is less than 4° in both cases.

### Generation and Detection of THz Radiation

**Figure 2:**
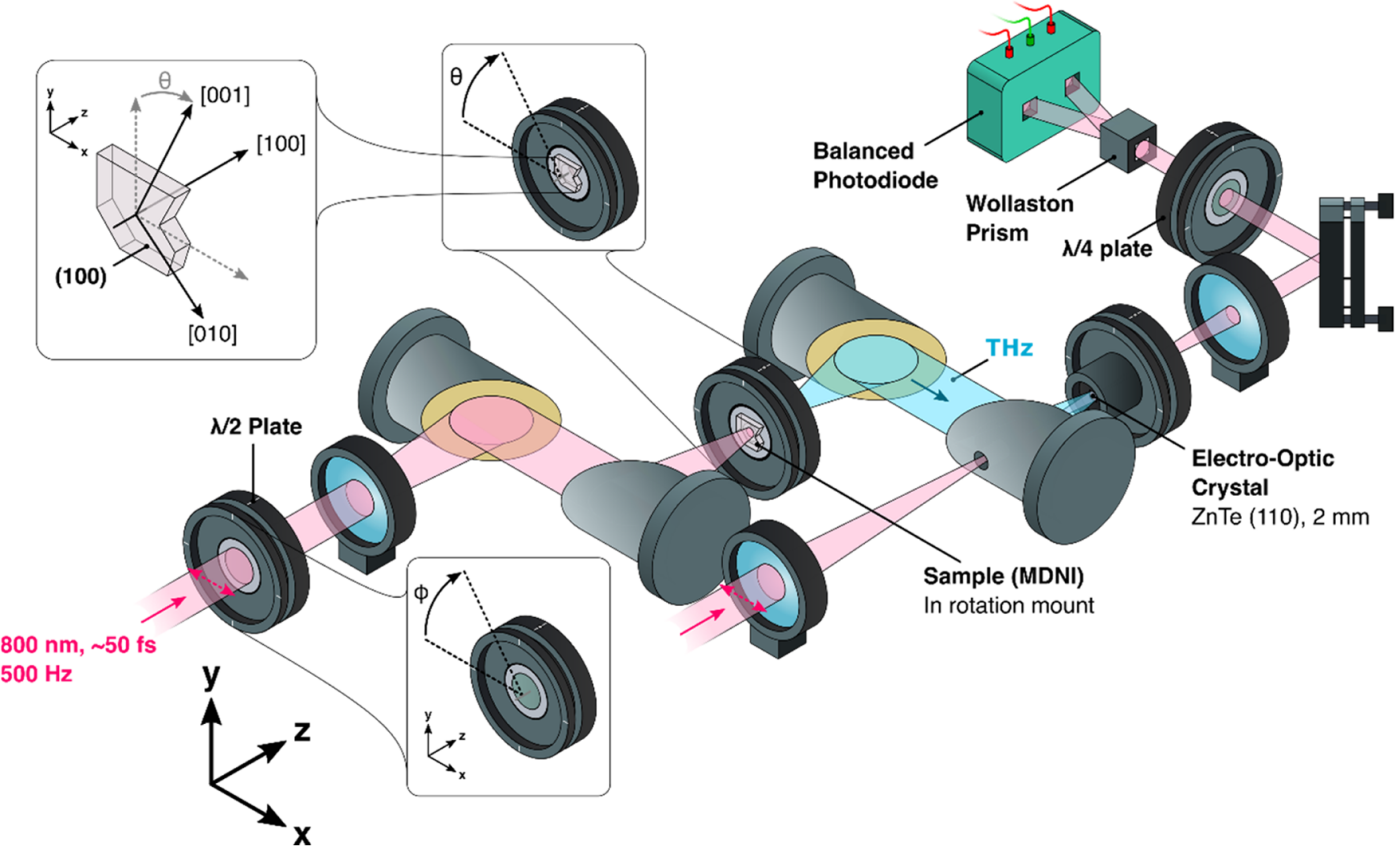
Illustration of the THz emission setup employed in this study. The 800 nm pump and probe beams are displayed in pink, while the THz output is displayed in blue. Solid arrows denote the direction of travel, while dashed arrows display the direction of polarization. The rotation of the sample and the λ/2 plate are given in the top right and bottom insets, respectively. The relationship between the orientation of the MDNI crystal, the crystallographic axes, and the laboratory coordinate system is displayed in the top left inset.

A schematic of the optical system employed for THz emission and detection is given in Figure 2. A 1 kHz Ti:sapphire regenerative amplifier (Spitfire Ace, Spectra Physics) was used to generate 800 nm pulses with a nominal width of ∼50 fs. Prior to generation of the THz field, a small portion of the 800 nm pulse was picked off for use in the electro-optical detection scheme.

To generate the THz pulses, the remaining 800 nm pulse (0.01–0.23 mJ cm^–2^) was focused onto a (100) cut MDNI crystal that was fixed to a rotating optic mount. The spatial FWHM of the 800 nm pulse at the sample surface was approximately 2 mm. As the polarization of the 800 nm input beam is highly relevant to the results discussed below, we optionally rotate the polarization of the pulse by means of a half-waveplate. Following generation of the THz field in the MDNI crystal, the THz pulses were separated from the transmitted 800 nm light by means of a nitrocellulose film. The electric field of the THz pulse was then characterized by means of a standard electro-optic detection scheme employing a 2 mm (110) cut ZnTe electro-optic detection crystal and a home-built balanced photodiode set. The entire THz beam path was enclosed in a purge box, and all measurements were taken in a nitrogen atmosphere.

### IR and THz Absorption of MDNI Crystals

IR spectra of MDNI in the 500–4000 cm^–1^ range acquired using a FTIR spectrometer (iS50R Nicolet, ThermoFisher Inc.) in the attenuated total reflectance (ATR) mode. IR spectra in the 80–400 cm^–1^ range were separately acquired in the reflectance mode using a Bruker 70 V FTIR spectrometer equipped with a Si beamsplitter and a DLaTGS detector. The MDNI crystals were ground to a fine powder in a pestle and mortar prior to measurement to obtain better contact with the ATR prism and, thus, improved absorption characteristics. To correct for imperfections in the ATR crystal and misalignment of the spectrometer, the obtained spectra were referenced against the spectrum of a gold mirror.

To obtain the THz time-domain spectrum (THz-TDS) of MDNI, we employed a standard lab-built THz-TDS setup based on a 100 fs Ti:sapphire oscillator with an 80 MHz repetition rate (Mai Tai, Spectra Physics). Briefly, the setup employs an interdigitated GaAs photoconductive emitter as the THz source. Detection of the THz radiation is performed using an electro-optical sampling scheme employing (111) cut GaP as the detection crystal. We obtained a THz-TDS spectrum on both an MDNI single crystal and an MDNI powder pellet. In the case of the MDNI single crystal, the crystal was polished to a thickness of approximately 400 μm to prevent excessive absorption of THz radiation. As with THz emission, the entire THz beam path was enclosed in a purge box, and all measurements were taken under a nitrogen atmosphere.

## Results

### THz Emission in MDNI

**Figure 3:**
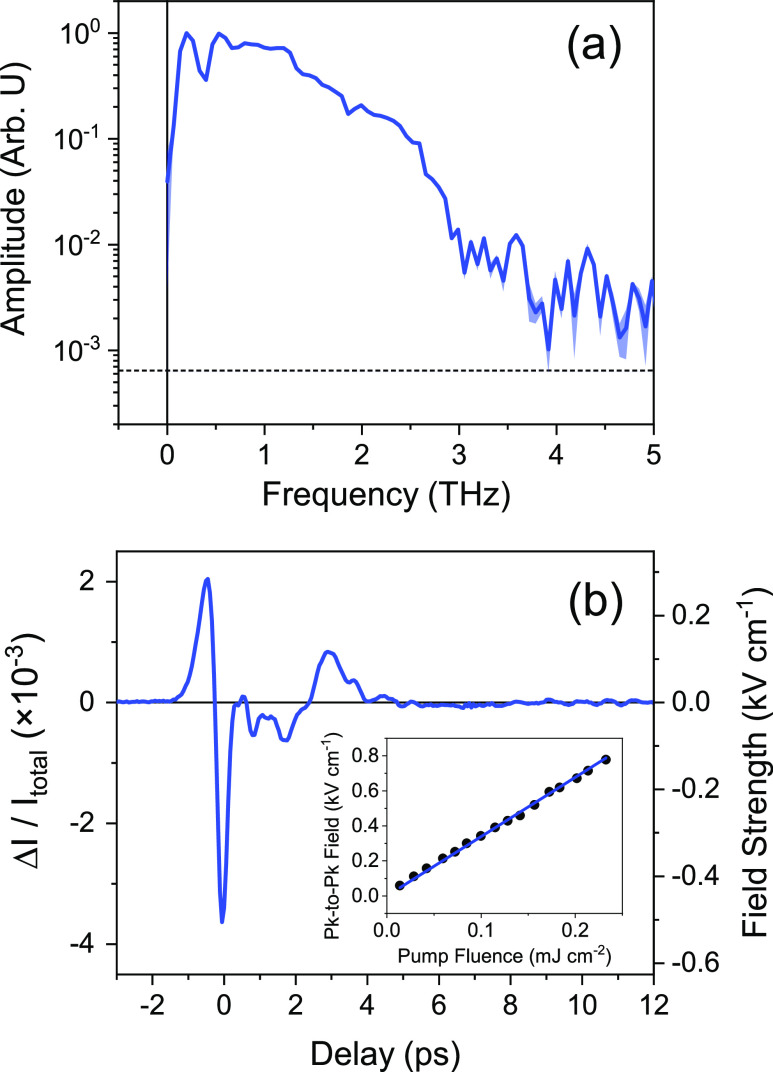
Characterization of the optimized THz field. (a) Spectrum of the emitted THz radiation; the shaded regions represent the standard deviation of 20 individual spectra, while the dashed line represents the noise floor of the instrument; the dynamic range of our detection system is approximately 5 × 10^4^ Arb. U (47 dB); (b) time-varying electric field of the THz pulse whose spectrum is given in (a) (inset: the dependence of the peak-to-peak field on the fluence of the 800 nm pump beam).

Figure 3 displays the optimized time and frequency domain signals of the generated THz pulse. Optimization of the observed signal was achieved through translation of the organic crystal along the direction parallel to the *z*-axis in Figure 2, rotation of the organic crystal and the λ/2 plate, and optimization of the overlap between the THz pulse and the probe beam. The pulse exhibits a broad spectrum (Figure 3(a)) from about 0.2–2.5 THz. Further emission beyond 2.5 THz is possible but not detectable given the bandwidth of the ZnTe detector crystal (See Supporting information (SI) Figure S1). Interestingly, the optimized spectrum exhibits only one moderate phonon resonance at approximately 0.4 THz, which compares favorably to other organic crystals.^8−12,34,35^ Rotating the crystal orientation, however (see Figure 5 and associated discussion, below) reveals additional phonon resonances that affect the temporal properties of the THz pulse.

The electric field profile of the THz pulse is given in Figure 3(b). The field profile is dominated by a derivative-like feature with an approximate temporal width of 900 fs. While the temporal profile of the THz field can be used in a small number of cases be used to exclude certain mechanisms of THz generation,^36,37^ the presence of intermediate optics between the MDNI and the ZnTe detector, along with dispersion of the THz pulse within the detection crystal, distorts the electric field. Thus, to characterize the mechanism of emission in the first instance, we varied the power of the input pulse and monitored the peak electric field of the emitted THz radiation. The resulting relationship between the pump intensity and the peak THz field is given in the inset of Figure 3(b), where a clear linear relationship between the peak field and the pump fluence can be observed. For THz radiation produced via optical rectification, the strength of the emitted THz field is expected to vary linearly with pulse fluence (See SI Section 1.1). Thus, the experimentally derived linear relationship given in Figure 3(b) is consistent with the emission of THz pulses via optical rectification.

We note that in other popular THz emitters, this expected linear relationship breaks down for large pulse fluences as two-photon absorption begins to compete with optical rectification processes;^38^ however the bandgap of MDNI (5.12 eV)^28^ is in the ultraviolet range and is over three times higher than the energy of an 800 nm photon (1.55 eV), making 2-photon and 3-photon absorption of the pump light negligible. By a similar token, the below-bandgap nature of our pump pulse also precludes the photogeneration of carriers necessary for THz emission via shift currents, whose overall amplitude would also be expected to vary linearly with intensity.^23,24,39^ Likewise, while the photo-Dember effect exhibits a nominally nonlinear dependence on the excitation intensity, it may approximate a linear relationship between the excitation fluence and peak THz field in regimes where the excitation fluence is significantly less than the intrinsic saturation fluence of the material,^40^ as has been observed with certain hybrid perovskite systems.^25^ However, as with shift currents, the photo-Dember effect requires the photogeneration of carriers in the material and is thus impossible, given the strongly off-resonant excitation conditions employed here. While it may be possible to stimulate these resonant processes in MDNI through multiphoton absorption, this would return a nonlinear dependence between the THz emission and pump fluence that is inconsistent with the experimentally obtained fluence dependence given in Figure 3(b).

Electro-optic sampling schemes such as the one employed in this study typically characterize the electric field of a THz pulse in terms of a dimensionless differential change in intensity (Δ*I*/*I*). In order to compare the THz emission of MDNI to other NLO materials, we calculate the total electric field strength of the THz pulse from the dimensionless differential intensity using the equation^41^
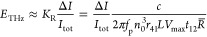
1where Δ*I/I*_tot_ is the difference in intensity between the two photodiodes normalized to the total intensity across both photodiodes, *f*_p_ is the center frequency of the probe pulse, *n*_0_ and *r*_41_ are respectively the linear refractive index and electro-optic coefficient of the detection crystal, *L* is the crystal length, *t*_12_ is the Fresnel coefficient at the air-crystal interface, *V*_max_ is a numerical factor that depends on the electro-optic crystal orientation, and and *R* is the average spectral response that depends on the spectral response function *R*(ω_THz_). This function accounts for the finite temporal width of the probe pulse, along with other effects, such as group and phase velocity mismatch between the THz pulse and the probe pulse. Both *r*_41_ and *R*(ω_THz_) depend on the frequency of the terahertz field and must be parametrized separately; we make use of parametrizations previously employed by Leitenstorfer et al. for *r*_41_ and *R*(ω_THz_),^42^ as discussed in SI Section 1. We further verified the calibration of our detection crystal by comparing it to a thinner gallium phosphide crystal, making use of a LiNbO_3_ emitter. Based on this analysis, we calculate the peak-to-peak electric field of mDABCO to be roughly 0.8 kV cm^–1^.

**Figure 4:**
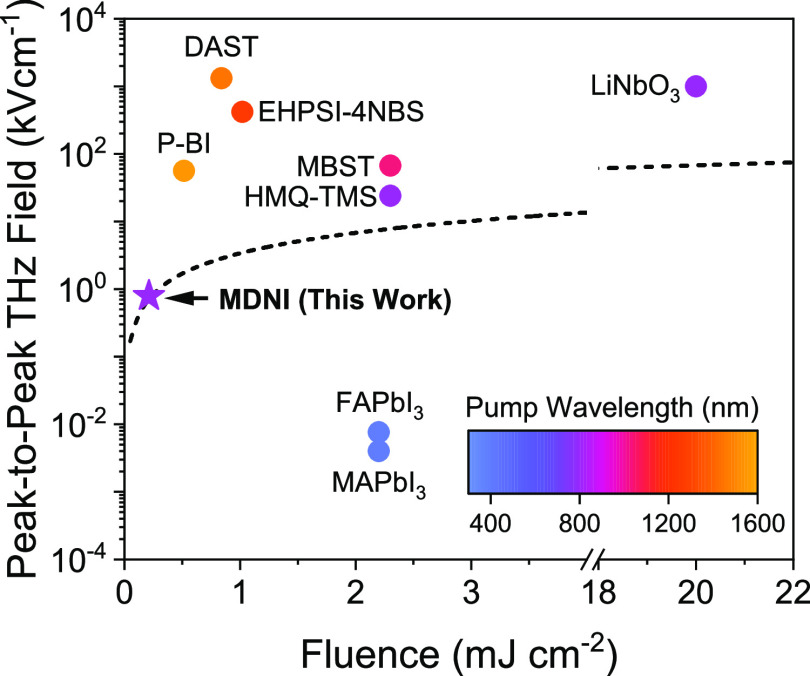
Comparison of MDNI to other recently reported THz emitters, including the high-performance organic emitter DAST, as well as MAPbI_3_ and FAPbI_3_. The dashed line is an extrapolation of the linear fit given in Figure 2(a). References for the various literature values used in the figure are supplied in the SI.

Figure 4 compares the performance of the MDNI emitter reported in this work to a variety of other organic crystalline emitters,^10,11,35,43^ as well as the common halide perovskite materials MAPbI_3_ and FAPbI_3_. Strikingly, we find that the THz emission of MDNI exceeds that of MAPbI_3_ and FAPbI_3_ by over 2 orders of magnitude despite the significantly higher fluence used for the latter materials. As compared to a standard, commercial spintronic THz emitter (T-Spin1, Teraspintec) used routinely for spectroscopic measurements in the same experimental setup, the emission from MDNI is only about a factor of 7 smaller (Table S2 and Figure S3). In fact, by extrapolating the linear trend given in the inset of Figure 3(b) we see that peak-to-peak fields on the order of 1–10 kV cm^–1^ are achievable by varying the pump fluence, with the emission of MDNI most closely matching the organic crystal 2-(4-hydroxy-3-methoxystyryl)-1-methilquinolinium-2,4,6-trimethylbenzenesulfonate (HMQ-TMS). Further optimization of parameters such as crystal thickness, pump wavelength, and the presence of a substrate beneath the emitter also provide opportunities to improve the performance of MDNI further still.^35^ Moreover, we note that MDNI exhibits high transparency in the visible and near-UV spectral regions—an issue that often plagues organic crystalline emitters, which would reduce multiphoton absorption processes at higher fluences and would enable more straightforward selection of excitation wavelengths compared to organic systems. The high transparency of MDNI likely contributes to its high (∼0.8 J cm^–2^) damage threshold when illuminated with ultrafast laser pulses,^28^ which is an order of magnitude higher than that of more standard hybrid perovskite materials and suggests that much stronger excitations than those used in this study could be employed in the future. Furthermore, the high bandgap of MDNI may facilitate better phase matching, as discussed in more detail in Section S4 in the SI.^51^

### Angular and Polarization Dependence of THz Emission in MDNI

We further characterized the emitted THz radiation and confirmed optical rectification as the operative THz generation mechanism by measuring its dependence on the rotation of the crystal, as well as the polarization of the incoming 800 nm pump pulse. For our experimental conditions, we outline a relationship for the effect of crystal orientation and pump polarization through the use of a Jones matrix equation (see SI Section 2), which is expressed as
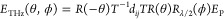
2where *E*_THz_ is a vector representing the strengths of the electric field in the *x*–*y* plane in the lab frame (see Figure 2); θ and ϕ are, respectively, the rotation of the MDNI crystal and the rotation of the half-wave plate; *R*(θ) and *R*_λ/2_(ϕ) are the matrices that account for polarization changes brought about by the half-waveplate; *T* is the projection matrix that maps the electric field to the crystallographic axes of the MDNI emitter; and *d*_*ij*_ is the second-order polarization tensor, which we employ alongside previously calculated values for the nonlinear susceptibility of MDNI. The exact matrix forms of these tensors are outlined in SI Section 2.

Two sets of angular dependencies were obtained by holding one set of rotational parameters (crystal rotation or polarization) constant while varying the other. In the case where the polarization of the pump pulse was rotated (Figure S6), the crystal was held at a rotation previously determined to yield the maximum THz signal, with the probe pulse held at the peak of the THz emission. We obtained excellent agreement between the experimentally obtained polarization dependence and the dependence calculated from eq 2, further confirming optical rectification as the THz generation mechanism.

**Figure 5:**
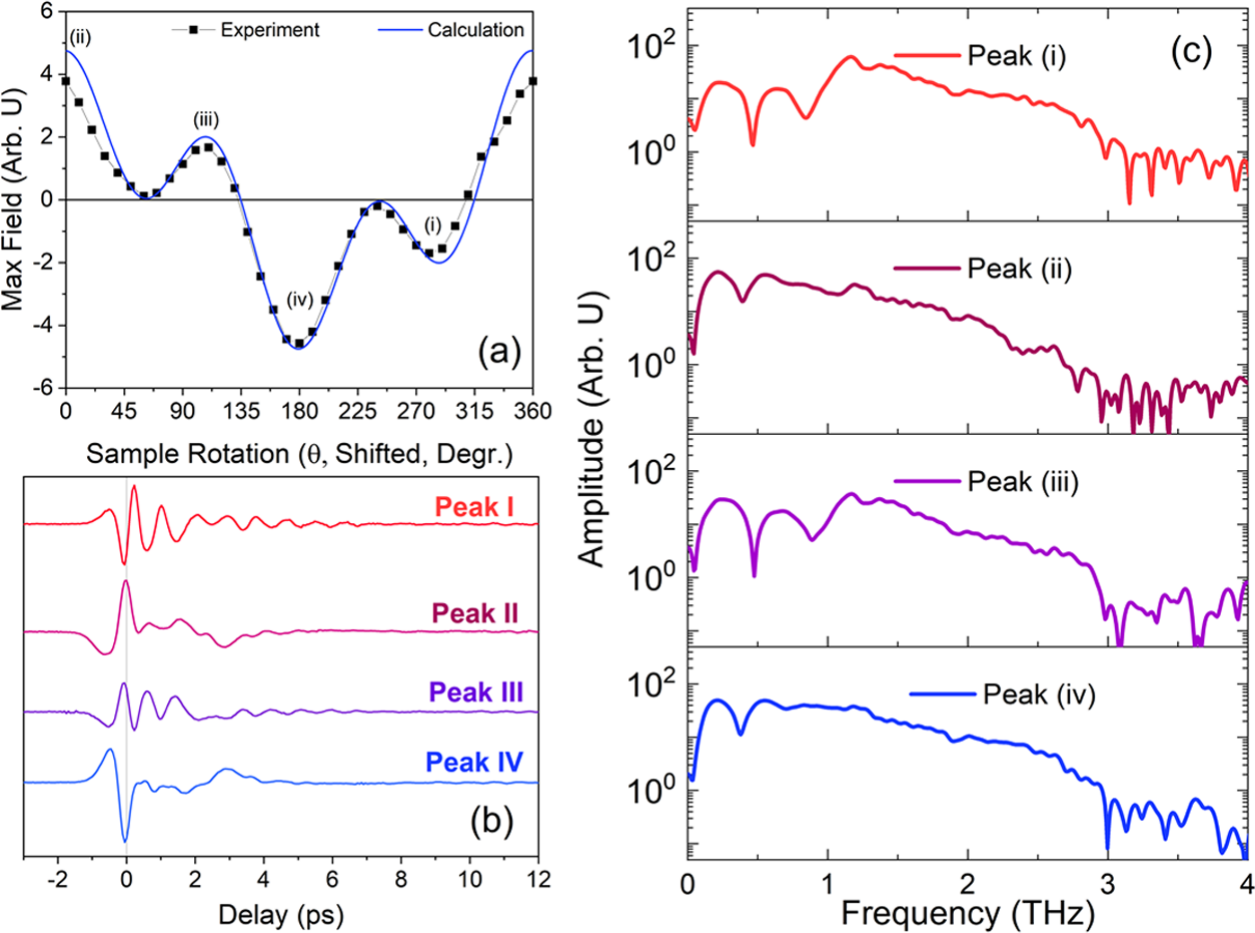
Rotational characterization of the THz emission. (a) Dependence of the THz emission on crystal orientation. The solid points represent the experimentally obtained strengths of the THz emission peak, while the solid blue line represents the predicted angular dependence of the peak field as predicted via eq 2. (b) THz waveforms of the four extremal points marked (I–IV) in panel (a). (c) Power spectra of the THz waveforms given in panel (b).

The relationship between the THz emission and crystal rotation is given in Figure 5(a). We note that previous calculations of the *d*_*ij*_ components of MDNI do not provide a value for *d*_31_, which is necessary to correctly calculate the angular dependence of emission from optical rectification; we therefore choose a value for *d*_31_ (2.48 pm V^–1^) that most closely agrees with the experimentally derived angular dependencies (see SI Section 2, for details). We obtained good agreement between the experimental and the calculated rotational dependencies. Slight deviations likely result from orientational dispersion of crystallites within the single crystal as well as from imperfections in the crystal cleaving process, resulting in inhomogeneities in thickness. Nevertheless, the qualitative agreement between the calculated THz output and observed THz emission further implicates optical rectification as the mechanism of THz generation in these materials.

We additionally note that the inclusion of a nonzero *d*_31_ element also implies that the performance of MDNI as a THz emitter is more promising than suggested by previous studies that evaluated MDNI as a nonlinear optical material for deep-UV second-harmonic generation.^28−30^ Although the obtained value of 2.48 pm V^–1^ is still an order of magnitude below that of other organic THz emitters,^34^ it is comparable to that of inorganic materials including GaAs and GaP.^48,49^

### Structural and Vibrational Characterization of MDNI

We additionally characterize the THz pulse at each of the four extremal points (marked (i)–(iv) in Figure 5(a)). We observe that for two of the extremal points (i) and (iii), the THz pulse exhibits a significant ringing component that persists for several picoseconds (Figure 5(b)). Spectra of the corresponding THz waveforms (Figure 5(c)) resolve this ringing component into a pair of sharp absorption lines at 0.4 and 0.8–0.9 THz, with an additional smaller absorption feature at approximately 1.3 THz. These lines suggest the existence of phonon resonances within the region of THz emission. Interestingly, while the resonance at 0.4 THz is present for all four orientational positions, the mode at 0.8 THz appears for only two orientations.

**Figure 6:**
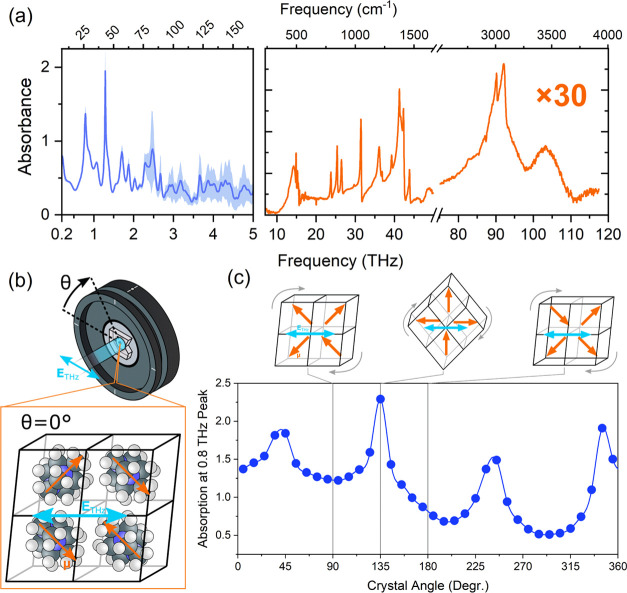
Vibrational properties of MDNI. (a) Vibrational spectrum of MDNI across the IR and THz spectral regions comprising a THz-TDS spectrum (blue line) and an IR-ATR spectrum (orange line). (b, c) Angular dependence of the ∼0.8 THz absorption line of MDNI: (b) Relationship between the rotation of MDNI in the lab frame, the crystallographic orientation of MDNI, the dipoles (orange arrows) of the [111] aligned mDABCO molecules, and the electric field of the polarized THz pulse (blue arrow); (c) the peak absorption of the ∼1 THz resonance as a function of crystal angle, including a proposed relationship between the crystal orientation, the orientation of the mDABCO moieties, and the electric field of the THz pulse that gives rise to the angular dependencies.

We further confirm the existence of these phonon resonances by obtaining both IR and THz-TDS spectra of an MDNI powder pellet, which is given in Figure 6(a). We identify two strong resonances at approximately 0.8 and 1.3 THz (approximately 27 and 43 cm^–1^), respectively, with weaker resonances observed in the 1.5–2.5 THz region and no significant resonances observed between 2.5 and 10 THz, which indicates that the THz emission of MDNI likely extends beyond the 2.5 THz bandwidth of our current detection system. The spectral locations and relative amplitudes of the two absorption lines at 0.8 and 1.3 THz agree well with the observed absorption lines in the emission of MDNI (Figure 5(c)). We additionally identify a rich array of vibrational resonances in the high-frequency (500–4000 cm^–1^) portion of the vibrational spectrum. Comparison of these features to previously reported IR spectra of DABCO and ammonium suggests that all of the observed bands in this region can be assigned to intramolecular modes of the methyl-DABCO and ammonium ions.

To assign the low-frequency bands at 0.4 and 0.8 THz, we compared the angular dependence of the THz absorption of MDNI to the rotational orientation of the MDNI crystal. By comparing the X-ray diffraction properties of our MDNI crystal to previously reported crystallographic properties, we establish that for θ ≈ 0, the polarization of our 800 nm pulse is coplanar with the [010] axis of the crystal (Figure 6(b)). We find that absorption at 0.8 THz peaks at four distinct angular positions. At these angles, we determine that the electric field of the THz pulse is roughly coplanar with both the [110] and [111] axes; this is notable as previous studies on metal-free perovskites have indicated that methyl-DABCO orients itself along the [111] crystallographic axis,^33,44,45^ which suggests that the mDABCO molecule is coplanar with the electric field of the THz pulses at these angles. Based on this, we suggest that the mode at 0.8 THz arises due to the linear displacement of the MDABCO ion within the cuboctahedral cavity.

Interestingly, we see no evidence of a mode at ∼0.4 THz, in our angularly resolved THz-TDS spectrum (Figure S) or in the THz-TDS spectrum given in Figure 6(a). We note both the angularly resolved THz-TDS spectra of the MDNI single crystal and the THz-TDS spectrum of MDNI powder were acquired using pristine MDNI crystals—the crystal had been freshly polished and the powder was newly synthesized. As a result, we tentatively assign the ∼0.4 THz mode of MDNI to a surface degradation product, potentially resulting from long-term exposure of the MDNI crystal to moisture. It should be noted that the MDNI crystal was stable in ambient conditions for a period of weeks in agreement with a previous report^50^ and in contrast to typical halide perovskites.

## Discussion

Our results clearly demonstrate the potential of MDNI as a material for THz generation. Encouragingly, we note that MDNI represents only one of a variety of potential all-organic perovskite species, and as such, the space of emitters that could be produced in the future is vast. In particular, studies on hybrid inorganic–organic perovskites have highlighted the degree to which the structural parameters of the material can be tuned depending on the material’s organic and inorganic constituents (for example, the structure of the perovskite can be tuned through alloying of organic A-site species or through the partial substitution of halide ions).^19,21,22,46^ This, when combined with the ability to alter the ionic species within the crystal, leads to a vast parameter space that allows for careful tuning of the material’s nonlinear optical properties and may also enable the use of highly polarizable organic species that would otherwise form inactive centrosymmetric crystals. Previous studies on organic NLO crystals have demonstrated the value of computationally screening a broad array of potential organic species;^12^ combining this technique with previously reported data mining approaches to screen combinations of organic, metal and halogen ions hybrid organic–inorganic perovskites could enable the rapid identification of potential crystal candidates.^19^ Moreover, our characterization of the THz absorbance of MDNI suggests that structural interplay between the methyl-DABCO moiety and the NH_4_I_3_ cage likely plays a significant role in dictating the nonlinear optical properties of MDNI. In MDNI, the attachment of a methyl group to the DABCO moiety breaks the symmetry of the lattice, effectively forcing the cation to adopt an acentric position within the surrounding cuboctahedral NH_4_I_3_ cage, in a manner broadly analogous to the noncentrosymmetricity of LiNbO_3_. Future studies that focus on the interplay between the methyl-DABCO moiety and the inorganic cage would also be beneficial for facilitating the rational design of future metal-free perovskite systems for nonlinear optics.

Driven by the desire for efficient methods of generating intense THz light, a considerable amount of effort has been directed toward the use of organic crystals as THz emitters.^34,35,47^ This involves both searching for new organic species for THz emission, as well as optimizing existing materials to make their use practical (for example, by improving damage thresholds).^35^ However, careful control of molecular packing is essential to ensure both a high number density and noncentrosymmetric arrangement of organic ions in these crystals. This is often problematic as molecular engineering techniques to improve the crystallinity and packing properties of promising organic materials can result in large and unpredictable changes to other properties of the material and *vice versa*,^34^ while other techniques to address issues of packing (such as poled polymer formation) necessarily come at the expense of optical performance.^34,47^ Here, the more predictable structural behavior of perovskite materials could aid in further improving the performance of organic NLO species; metal-free perovskites are able to make use of organic small molecules within their crystal structure, which opens up the possibility of introducing highly polarizable functional groups to enhance the nonlinear properties of the material further.^29^ We note that ab initio calculations of the optical properties of other of metal-free perovskites suggest that that this is a promising approach to take.^29^ Meanwhile, further optimization of parameters such as crystal thickness, pump wavelength, and the presence of a substrate beneath the emitter also provide opportunities to improve the performance of MDNI further still.^35^

In conclusion, we report on the first of a potentially new family of materials for THz emission. Illumination of our emitter, methyl-DABCO DNI (MDNI) with ultrafast 800 nm pulses results in the emission of THz radiation with a bandwidth of >2.5 THz. Characterization of the emission implicates optical rectification as the mechanism of THz pulses. Our results demonstrate the ability of this new family of all-organic perovskite crystals to serve as organic crystalline THz emitters.

## Supporting Information Available

Calculation of the THz field intensities; calculation of the angular dependencies using the Jones matrix formalism; additional characterization of MDNI; and comparison of THz emission of MDNI to other crystals (PDF)
