# Supplementary material for: Terahertz Emission via Optical Rectification in a Metal-Free Perovskite Crystal
Source: ACS Photonics. 2023 Oct 18;10(11):4022–30. doi: 10.1021/acsphotonics.3c00918 (PMC10655262; doi:10.1021/acsphotonics.3c00918)
Supplement: Supplementary file 1 — ph3c00918_si_001.pdf [file ph3c00918_si_001.pdf]

Supplementary Information for: Terahertz Emission via Optical Rectification in a Metal-Free Perovskite Crystal

Nathaniel P. Gallop,[1] Dumitru Sirbu,[2] James Lloyd-Hughes,[1] Pablo Docampo,[2,3] and Rebecca L. Milot[1]

1. *Department of Physics, University of Warwick, Coventry, CV4 7AL, United Kingdom*
2. *Department of Chemistry, University of Newcastle, King's Rd, Newcastle, NE1 4LB, United Kingdom*
3. *School of Chemistry, University of Glasgow, Glasgow, G12 8QQ, United Kingdom*

Contents

1. Calculation of THz Field Intensities S2
   1.1 Linear Dependence of Optical Rectification S2
   1.2 Calculation of THz Field Strengths S2
   1.3 Referencing of ZnTe Crystal S4
2. Calculation of Angular Dependencies Using Jones Matrix Formalism S6
3. Characterisation of MDNI crystals S10
   3.1 Dynamic Range of the THz Detection System S10
   3.2 IR and THz-TDS spectra of MDNI S10
   3.3 Structural Characterisation of MDNI S11
4. Comparison of MDNI to Other Crystals S12
5. References S13

1. Calculation of THz Field Intensities

1.1 Linear Dependence of Optical Rectification

For THz fields generated *via* optical rectification, the field strength of the emitted THz pulse is proportional to the nonlinear polarization of the medium, which is related to the field strength of the excitation pulse ($E_0(t)$) by:

$$E_{THz} \propto P_{NL}(t) = \sum_{j,k} \varepsilon_0 \, \chi^{(2)}_{ijk} \, E_j(t) E_k(t) \tag{S1}$$

where $P_{NL}(t)$ is the nonlinear polarisation, $\chi^{(2)}$ is the second-order nonlinear susceptibility of the medium, and ε_0 is the permittivity of free space.

For a pulse of light, the total energy density (fluence) is directly related to the electric field strength *via*:

$$I_p = \frac{\varepsilon_0}{2} |E_0(t)|^2 \tag{S2}$$

where I_p is the fluence. A relationship between I_p and E_{THz} follows from the combination of Eqns. S1 and S2:

$$E_{THz} \propto I_p \tag{S3}$$

This establishes a linear relationship between the fluence of our pump pulse and the electric field of the THz pulse.

1.2 Calculation of THz Field Strengths

To convert the arbitrary values of the THz waveforms to standard SI values, we make use of the formalism outlined by van der Valk *et al.*[1]. For an electro-optic sampling scheme employing a perfectly transparent and dispersion-free electro-optic sampling crystal, the relative change in intensities at the two photodiodes ($^{\Delta I}/_{I_{tot}}$) in the limit of low THz fields is given by:

$$^{\Delta I}/_{I_{tot}} = \frac{\omega_p \Delta n L}{c} = \frac{\omega_p n_0^3 r_{41} L}{c} (\boldsymbol{E_{THz}} \cdot \boldsymbol{V}) \tag{S4}$$

where $\boldsymbol{E_{THz}}$ is a vector containing the electric field components of the THz pulse, ω_p is the center angular frequency of the probe pulse, n_0 is the refractive index of the detector material at the probe wavelength, L is the length of the crystal, and r_{41} is the electro-optic coefficient. The vector $\boldsymbol{V}$ accounts for the direction of propagation of the probe beam, as well as the polarization of the probe beam relative to the crystallographic axes of the electro-optic detection crystal and is described in greater detail elsewhere.[1] In practice, the length and direction of $\boldsymbol{V}$ can be optimized by rotating the electro-optic crystal; assuming the polarization of the THz field is collinear with that of the probe field and that the crystal orientation has been rotated to maximize the observed THz field, $\boldsymbol{V}$ reduces to a single numerical factor that depends solely on the crystallographic orientation of the electro-optic crystal, which we denote V_{max}. Thus, the magnitude of the THz field (E_{THz}) along the direction collinear to the polarization of probe pulse is given by:

$$\Delta I /_{I_{tot}} = \frac{\omega_p n_0^3 r_{41} L\, V_{max}}{c} E_{THz} \tag{S5}$$

This relationship assumes an ideal electro-optic crystal, in which reflection of the THz pulse at the air-crystal interface, along with absorption of the visible and THz beams within the crystal, are negligible. To account for reflection losses at the air-crystal interface, we include a Fresnel factor (t_{12}) that depends on the refractive index of the electro-optic crystal in the THz region:

$$t_{12} = \frac{2}{n(\omega_{THz}) + 1} \tag{S6}$$

Moreover, Eqn. S2 correctly returns the electric field strength if and only if r_{41} is frequency independent. In general, this assumes the the phase velocity of the terahertz pulse at each frequency matches the group velocity of the probing pulse, which may not hold for real materials. For our choice of detection crystal (2 mm (110)-cut ZnTe) within the observed ~2.5 THz detection bandwidth of our detection crystal, r_{41} varies smoothly from approximately 4×10^{-12} m/V to approximately 3.8×10^{-12}, as such, we take an average value of 3.9×10^{-12} m/V for r_{41}. We additionally must include a normalized response function ($R(\omega_{THz})$) that accounts for the finite pulse width of the probe pulse, along with other effects, such as group and phase velocity mismatch between the THz pulse and the probe pulse. From these parameters, the terahertz field can then be expressed in terms of the relative change in intensity at the balanced photodiode as:

$$E_{THz} = \Delta I /_{I_{tot}} \frac{c}{\omega_p n_0^3 r_{41} L\, V_{max}\, t_{12}\, R(\omega_{THz})} \tag{7}$$

Alongside r_{41}, the response function $R(\omega_{THz})$ is also dependent on the frequency of the emitted THz radiation. To compute the response, we make use of parameterisations employed by Gallot *et al.*[2]. The resulting un-normalised response function for a selection of ZnTe crystals is given in Fig. S2, below.

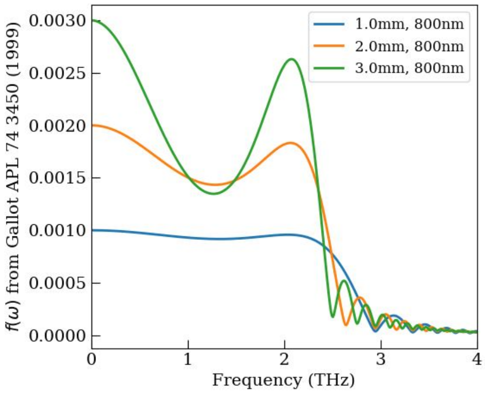

Figure S1. Calculated response function ($R(\omega_{THz})$) of ZnTe for three crystal thicknesses

As with the electro-optic coefficient, within the bandwidth of the THz emission, $R(\omega_{THz})$ varies smoothly and thus for ease of calculation, we replace the frequency dependent response function (ω_{THz}) with an average value $\bar{R}$, thus:

$$E_{THz} \approx \Delta I /_{I_{tot}} \frac{c}{2\pi\, f_p\, n_0^3 r_{41} L\, V_{max}\, t_{12}\, \bar{R}} \equiv K_R \times \Delta I /_{I_{tot}} \tag{S8}$$

Where K_R is a constant of proportionality that converts the differential signal at the photodiodes to the electric field strength. For additional ease of calculation, we have replaced the angular frequency (ω_p) with a matching expression of the absolute frequency of the probe pulse (f_p). The relevant constants for the calculation of K_R are given in Table 1:

Table S1. Parameters employed for calculation of K_R

Parameter	Definition	Value (ZnTe)	Value (GaP)	Units
$\boldsymbol{c}$	Speed of Light	3.00 × 10^8	3.00 × 10^8	ms^{-1}
$\mathbf{f}_{\boldsymbol{p}}$	Probe centre Frequency	374.74 × 10^{12}	374.74 × 10^{12}	s^{-1}
$\boldsymbol{n_0}$	Refractive index at probe frequency	2.85	3.19	None
$\boldsymbol{n(\omega_{THz})}$	Refractive index in THz range	3.20	3.30	None
$\boldsymbol{r_{41}}$	Electro-optic coefficient	3.9 × 10^{-12}	0.5 × 10^{-12}	m V^{-1}
$\boldsymbol{L}$	Electro-optic crystal thickness	0.002	0.0002	M
$\boldsymbol{\bar{R}}$	Average response function	0.79	1	None
$\boldsymbol{V_{max}}$	Crystal orientation factor	1	$\sqrt{6}/3$	None
$\boldsymbol{t_{12}}$	Fresnel Coefficient	0.48	0.47	None
$\boldsymbol{K_R}$	**THz Conversion Factor**	**14.8**	**1032**	**kV cm^{-1}**

1.3 Referencing of ZnTe Crystal

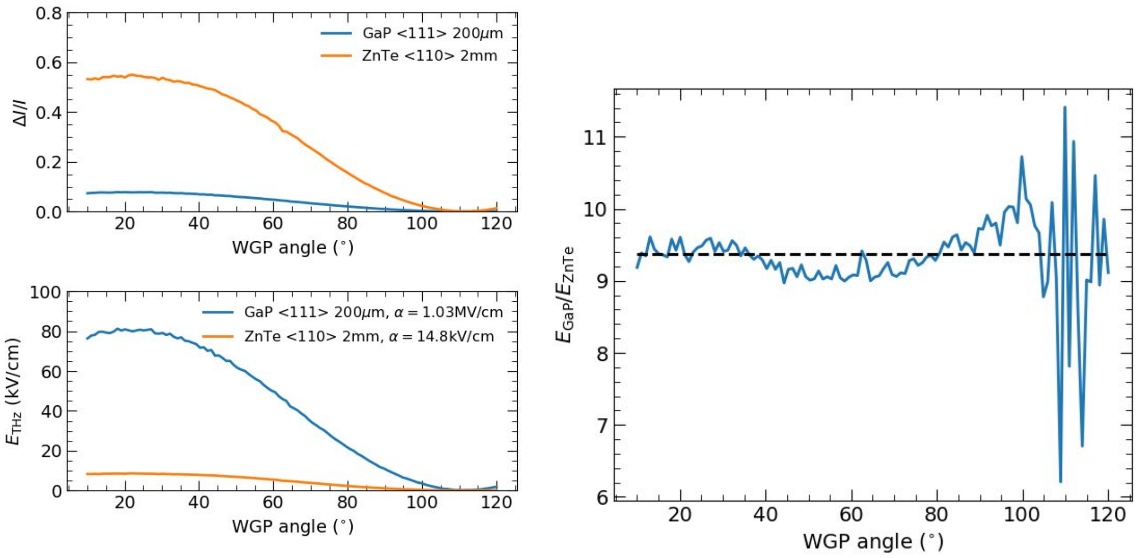

Figure S2. Referencing of ZnTe crystal against GaP crystal. (left) Peak THz field of an $LiNbO_3$ emitter measured using the <110> cut ZnTe crystal employed in this study (orange curve), and a 200 μm <111> cut GaP detection crystal. The emitted THz field was attenuated using a wire-grid polariser. The output of the $LiNbO_3$ emitter is identical across the two measurements and thus the detected peak THz field should also be identical. (right) Ratio between the peak THz field amplitudes sampled using the ZnTe and GaP crystals; the ratio between the measured fields is consistent as a function of polariser angle (and hence, THz intensity) at around 9.4.

Finally, it is necessary to account for nonidealities arising from absorption losses and other factors (*e.g.:* crystal strain, surface imperfections, *etc.*) within our crystal. In practice, it is impossible to find simple mathematical expressions to account for these non-idealities—instead we account for these factors by comparing the performance of our ZnTe crystal with that of a 200 μm, (111) cut GaP crystal, whose electro-optic parameters are also given in Table 1, above. We then characterize the emission

of an $LiNbO_3$ crystal using both our ZnTe detection crystal and the GaP detection crystal. We control the power of the resulting THz emission using a wire grid polarizer.

The uncalibrated and calibrated THz peak amplitudes are given in Fig. S2 Given that we are characterizing the same THz generation system, we expect the calibrated response of our two detection systems to return identical THz field strengths (to within a factor of the shot-to-shot variability of the 800 nm excitation laser). However, we find that the calibrated THz field strength detected using the GaP crystal is approximately nine times greater than when detecting a virtually identical field using ZnTe. Given the thickness of the GaP crystal is roughly 1/10th that of the ZnTe crystal, this suggests that parasitic absorption losses within the ZnTe crystal are strongly attenuating the THz pulse as it passes through the material. These internal losses mean that the true electric field of the THz pulse emitted by the MDNI system is roughly 9.4 times greater than the detected by the GaP. Correcting for this effect means that the 'effective' K_R value for our chosen ZnTe crystal is **139.1 kV cm^{-1}.**

Table S2. Parameters used to calculate the peak-to-peak THz emission for MDNI and a spintronic emitter.

Emitter	ΔI	I_{tot}	$\Delta I/I_{tot}$	Peak to Peak Field (kV cm^{-1})
**MDNI**	8.84	1580	0.0057	**0.778**
**Spintronic**	66	1580	0.04	**5.56**

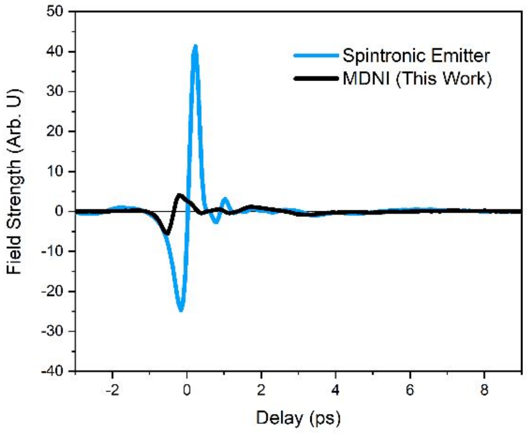

Figure S3. THz emission from MDNI and a spintronic emitter taken under the same conditions.

2. Calculation of Angular Dependencies Using Jones Matrix Formalism

To obtain the angular dependence of the emitted THz electric field, it is necessary to understand how the polarization of the pump and THz emission change as they propagate through the optical train displayed in Fig. 2 (Main Text). The polarization of the THz field is related to the polarization of the pumping field by the following matrix equation:

$$E_{THz} = R(-\theta)\, T^{-1}PT\, R(\theta)\, R(-\phi)W_0R(\phi)\, E_P, \tag{S9}$$

where $R(\phi)$ and $R(\theta)$ are respectively the rotation matrices that account for the rotation of the half wave plate and the MDNI crystal, T is a projection matrix that maps the electric field in the laboratory frame of reference to the crystallographic co-ordinates of the MDNI crystal, P maps the electric field of the traversing pulse to the polarisation of the crystal, W_0 accounts for the phase difference of the pump pulse along the ordinary and extraordinary axes of the half-wave plate, and E_{THz} and E_P are respectively the vector representations of the THz field and the pump pulse.

We begin with a pump pulse that is linearly polarised along the x-axis in the laboratory frame, the vector representation of the pulse is therefore given by:

$$E_P = \begin{pmatrix} E_P \\ 0 \\ 0 \end{pmatrix}. \tag{S10}$$

Considering first the traversal of the pump pulse through the half-wave plate, the form of W_0 is given by the relevant Jones matrix[3]:

$$W_0 = \begin{pmatrix} e^{i\pi/2} & 0 & 0 \\ 0 & e^{-i\pi/2} & 0 \\ 0 & 0 & 1 \end{pmatrix}. \tag{S11}$$

Importantly, this form W_0 only applies when the polarisation of the incoming field is aligned to the optical axis of the waveplate. To account for rotation of the optic, it is necessary to include two rotation matrices that map the field in the lab frame to optical axes of the waveplate and then map the rotating electric field back to the laboratory frame. This is accomplished by the two rotation matrices $R(+\phi)$ and $R(-\phi)$. Rather than account for each of these processes separately, it can be convenient to take the product of the three matrices and use the resulting parameter instead, this gives:

$$R(-\phi)W_0R(\phi) = \begin{pmatrix} \cos\phi & \sin\phi & 0 \\ -\sin\phi & \cos\phi & 0 \\ 0 & 0 & 1 \end{pmatrix} \begin{pmatrix} e^{i\pi/2} & 0 & 0 \\ 0 & e^{-i\pi/2} & 0 \\ 0 & 0 & 1 \end{pmatrix} \begin{pmatrix} \cos\phi & -\sin\phi & 0 \\ \sin\phi & \cos\phi & 0 \\ 0 & 0 & 1 \end{pmatrix} \tag{S12}$$

Ignoring prefactors, this gives:

$$R_{\lambda/2}(\phi) \equiv R(-\phi)W_0R(\phi) = \begin{pmatrix} \cos 2\phi & \sin 2\phi & 0 \\ \sin 2\phi & -\cos 2\phi & 0 \\ 0 & 0 & 1 \end{pmatrix} \tag{S13}$$

Having modelled the rotation of our pulse polarization, we then project the polarization along the crystallographic axes of our MDNI crystal. The relationship between the axes in the lab frame and the crystallographic axes is given in the inset of Fig. 2 (main text). We use these axes to define our transfer matrix, T as:

$$T = \begin{pmatrix} 0 & 1 & 0 \\ 1 & 0 & 0 \\ 0 & 0 & 1 \end{pmatrix} \tag{S14}$$

Finally, the form of our second-order nonlinear tensor, d_{ij} is defined by the space group of the crystal. For MDNI, which adopts the R3 space group, d_{ij} takes the form[4]:

$$d_{ij} = \begin{pmatrix} d_{11} & -d_{11} & 0 & d_{14} & d_{15} & -d_{22} \\ -d_{22} & d_{22} & 0 & d_{15} & -d_{14} & -d_{11} \\ d_{31} & d_{31} & d_{33} & 0 & 0 & 0 \end{pmatrix} \tag{S15}$$

We employ the d_{ij} coefficients previously obtained *via ab initio* calculations by Kasel *et al.*, which are summarized in the table below.

Table 3 Values of the nonlinear optical coefficients for MDNI obtained by Kasel et al.

**d-coefficient**	d_{xxx}	d_{xyz}	d_{xxz}	d_{yyy}	d_{zxx}	d_{zzz}
**Contracted Representation**	d_{11}	d_{14}	d_{15}	d_{22}	d_{31}	d_{33}
**Value (pm/V)**	0.140	0	0.430	-1.200		0.021

We note that these calculations were conducted in the context of SHG, which enables a number of simplifying assumptions to be applied, specifically that $d_{31} = d_{15}$, and that $d_{14} = 0$. These assumptions, collectively referred to as Kleinman symmetry, result in a contracted form (d_{ij}') of the nonlinear optical tensor:

$$d_{ij}' = \begin{pmatrix} d_{11} & -d_{11} & 0 & 0 & d_{15} & -d_{22} \\ -d_{22} & d_{22} & 0 & d_{15} & 0 & -d_{11} \\ d_{15} & d_{15} & d_{33} & 0 & 0 & 0 \end{pmatrix} \tag{S16}$$

We note that exploiting Kleinman symmetry is only valid when the dispersion of the nonlinear susceptibility of the material is negligible. In cases such as SHG, this can represent a valid assumption; here it is likely inapplicable given the extremely large difference in the frequencies (~10^2 THz) of the 800 nm excitation pulses and the output THz pulse. We further demonstrate this by comparing the experimentally derived dependence on the orientation of the MDNI crystal to the dependence predicted using Eqn. S5, along with the form of d_{ij}' given in Eqn. S12. The calculated curve, displayed in Fig. S3, is in poor agreement with the experimentally obtained data. As a result, we resort to the full form of d_{ij} given in Eqn. S11. We note that the previously obtained d_{ij} coefficients do note specify a value for d_{31} (d_{zxx}), which appears in Eqn. S11 but not in Eqn. S12. We estimate a value for d_{31} by comparing the shape of the experimentally obtained and calculated angular dependencies for a variety of d_{31} values (Fig. S4); a similar technique which has been employed previously obtain values for the nonlinear susceptibility tensor in other materials.[5,6] In particular, we find that altering the value of d_{31} alters the amplitude ratio of the 'large' and 'small' peaks, which we label P_2 and P_1, respectively. By plotting the ratio of these two peaks and finding the value that best matches the experimentally determined peak ratio, we obtain a value for d_{31} of approximately 2.48 pm/V.

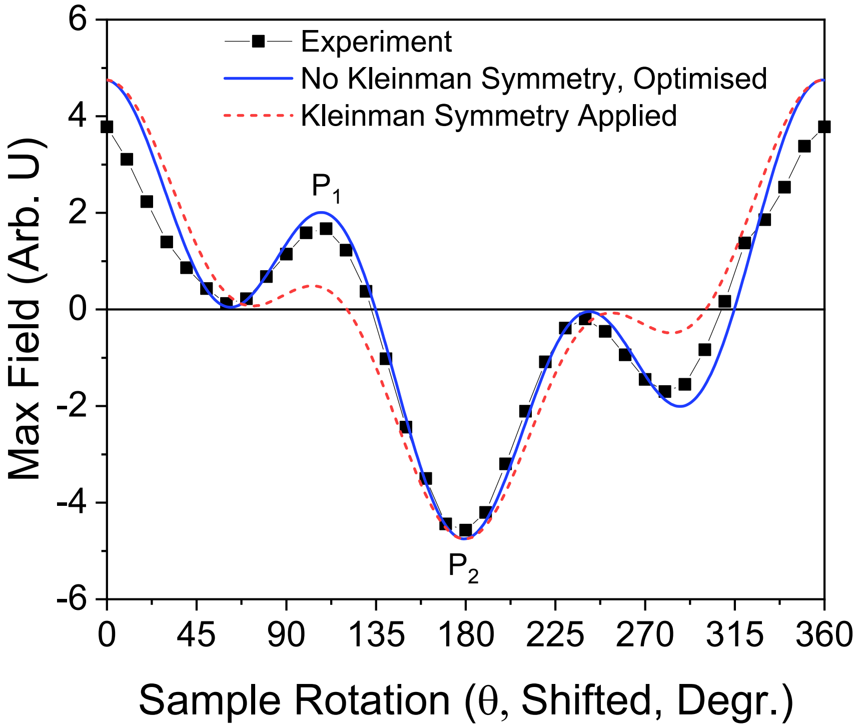

Figure S4. Comparison of calculated THz emission properties with (dashed red line) and without (solid blue line) the application of Kleinman symmetry. The peaks P1 and P2 are used to optimise the calculated curve to obtain agreement with experiment.

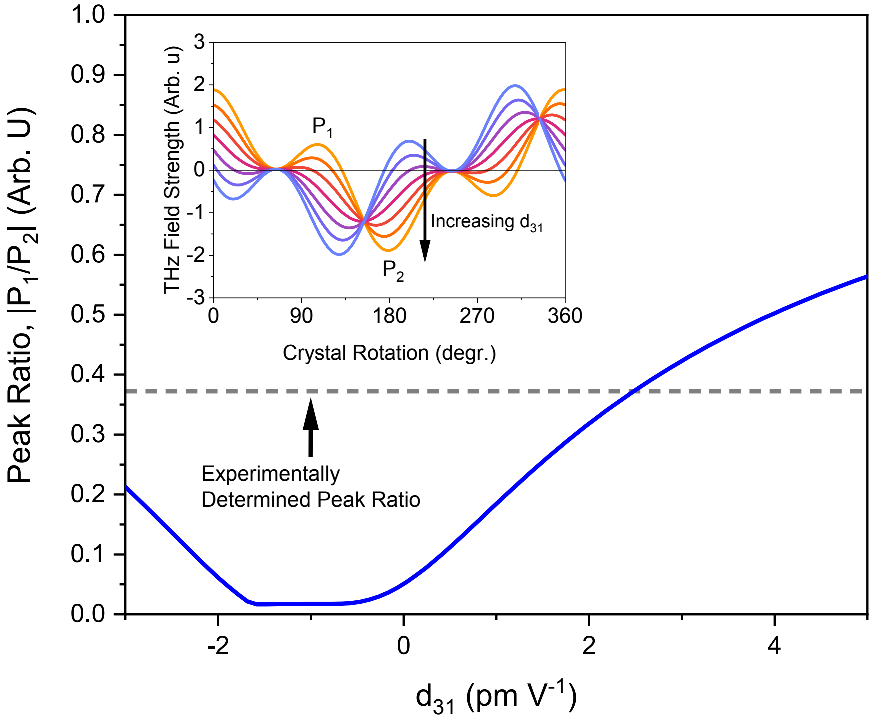

Figure S5. Ratio of P2 and P1 (See figure S2, above) as a function of d_{31} (inset: a series of angular dependency curves illustrating the effect of altering d_{31}).

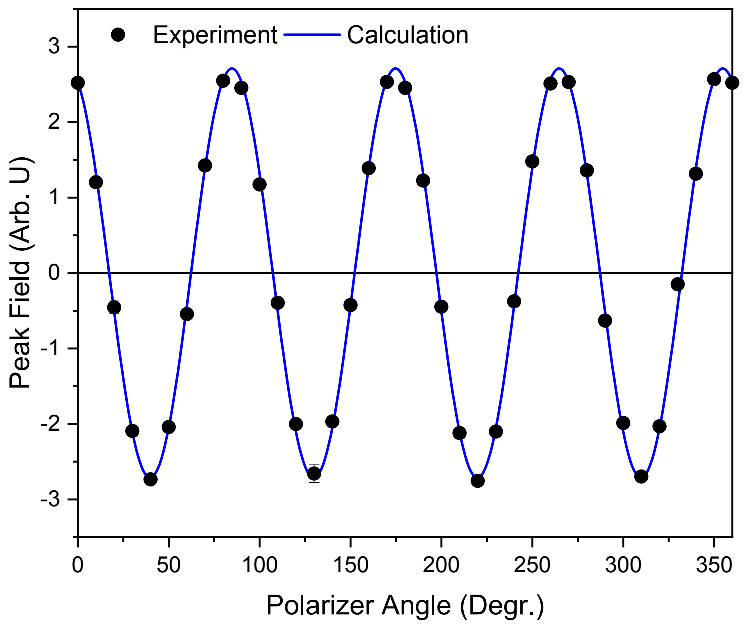

Figure S6. Dependence of MDNI emission as a function of pulse polarisation. Experimentally obtained field strengths are displayed as black points, whilst the calculated polarisation of The polarisation was altered via *the half-wave plate shown in Fig. 2 (main text). The expected response was calculated using SI Eqn. 4, holding ϑ constant and allowing φ to vary.*

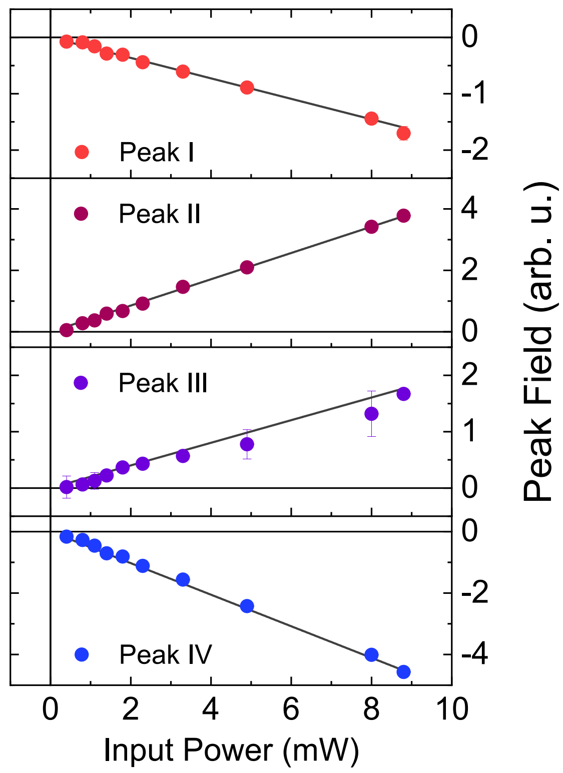

Figure S7. Power dependence of MDNI emission at the four extremal points marked I—IV, as defined in main text Fig. 5(a).

3. Characterisation of MDNI crystals

3.1 Dynamic Range of the THz Detection System

To characterize the dynamic range of our THz detection system, we obtained the spectrum of a THz pulse produced by an inverse spin hall effect emitter (Fig. S7, below). To estimate the dynamic range of our instrument, we obtain a noise floor by blocking the THz beam path with a metal block and taking the resulting spectrum. The spectrum of the spin Hall effect system with and without the beam block are given below:

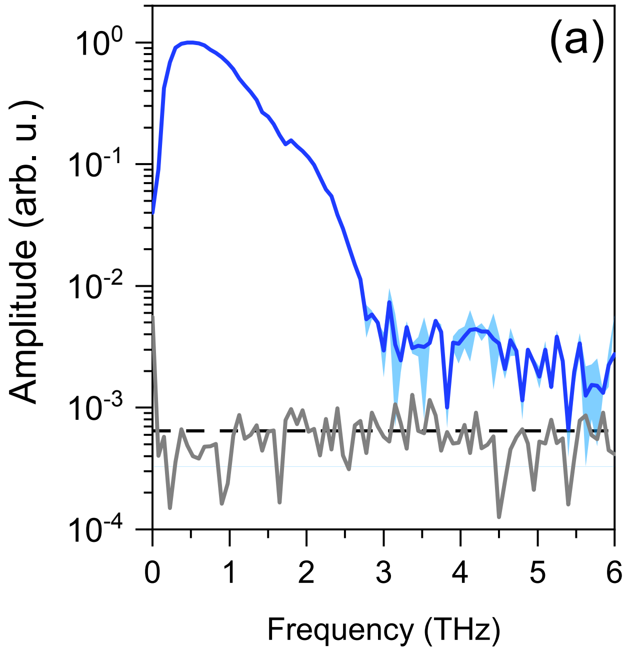

Figure S8. Spectrum of the THz pulse (blue line) produced using the spintronic emitter in the absence of an MDNI crystal; the noise floor of our detection system (grey line) is also given, with the average noise level demarcated by the dashed line.

Based on these measurements, we estimate that the dynamic range of our THz emission system is roughly 5×10^4 Arb. U (47 dB).

3.2 IR and THz-TDS spectra of MDNI

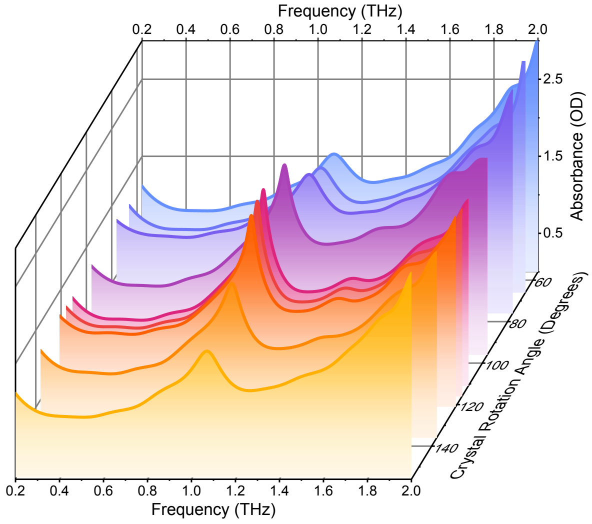

Figure S9. THz absorbance of MDNI as a function of crystal rotation angle for values of ϑ between 60 and 150 degrees.

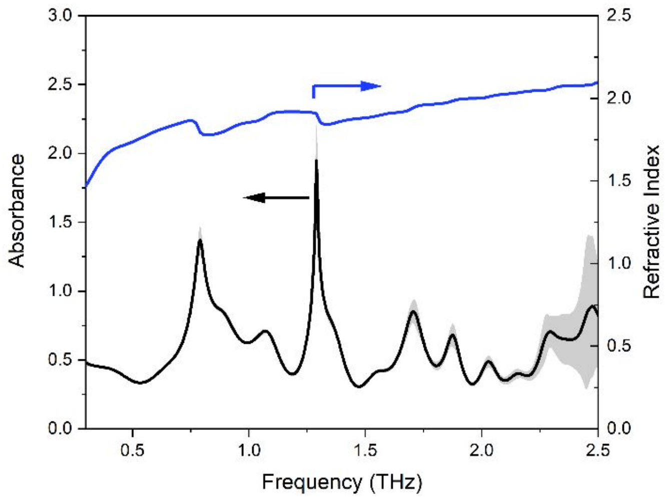

Figure S10. Absorbance and refractive index determined for the MDNI powder.

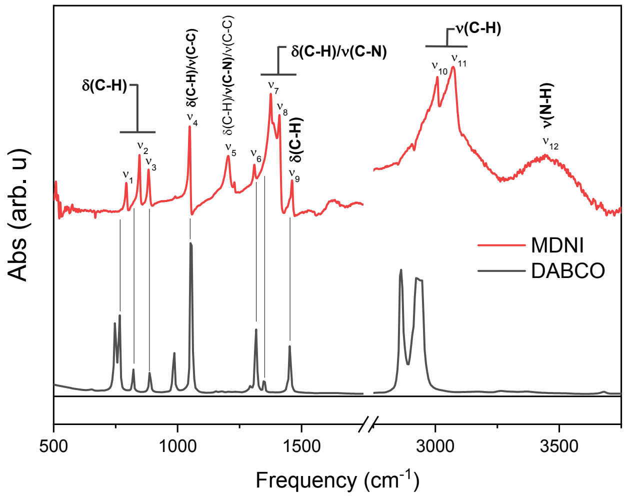

Figure S11. Comparison of the ATR spectrum of powdered MDNI (red lines) and DABCO (grey lines). Assignments of the IR bands of MDNI are based on comparison of the IR bands to those of MDNI. The IR spectrum of DABCO was obtained from the NIST webbook.[7]

3.3 Structural Characterisation of MDNI

To confirm the orientation of our MDNI crystal in the laboratory frame, we acquired single crystal XRD patterns of MDNI. To characterise the misorientation of our crystal and to establish the primary and secondary miller indices, we compared our obtained 2θ values for the [102] and [200] peaks with their expected positions, using a previously reported structure for the R3 phase of MDNI (a=b=c=7.259 A, α=β=γ=84.747°). Based on this analysis, we find the misorientation of the primary and secondary miller indices is less than 4° in both cases.

4. Comparison of MDNI to Other Crystals

We compare the performance of MDNI to other crystals in Fig. 5 (main text). The cited performances are given in Table S4 below. Where possible, sources were chosen where the fluence, excitation wavelength and peak-to-peak field were known. In cases where the peak-to-peak field wasn't explicitly given, the field was estimated *via* digital extraction of the time-domain curve from a suitable figure in the reference.

Table S4. Comparison of peak-to-peak field strengths of organic and perovskite THz emitters.

Crystal	Peak-to-Peak field (kV/cm)	Pump Wavelength (nm)	Pump Fluence (mJ cm^{-2})	Reference
**MDNI**	0.778	800	0.01—0.2	**This Work**
**EHPSI-4NBS**	133—417	1250	0.22—1.02	8
**P-BI**	22—56	1550	0.218—0.516	9
**MBST**	24	800	2.3	10
**HMQ-TMS**	67	800	2.3	10
**DAST**	1316	1450	0.84	11
**$LiNbO_3$**	375	375	1.5	12
**$MAPbI_3$**	4×10^{-3}	395	2.2	13
**$FAPbI_3$**	7.6×10^{-3}	395	2.2	14

The efficiency of the THz emission from a given crystal with a finite length is also dependent on the phase matching between the THz and optical pulses. One metric used to characterize phase matching is the coherence length l_c, which is estimated as follows:[15]

$$l_c = \frac{\lambda_{THz}}{2\left(n_{THz} - n_{pump}\right)} = \frac{c}{2\nu_{THz}\Delta n},$$

where λ_{THz}, ν_{THz}, and n_{THz} are the wavelength, frequency and refractive index of the THz radiation, n_{pump} is the refractive index of the optical pump, and *c* is the speed of light. For MDNI, we determined n_{THz} to be approximately 2 at a frequency of 1.5 THz. Although there are no known reports for the refractive index of MDNI at the 800 nm pump frequency, it can be estimated since the refractive index of perovskites has been shown to be dependent on the bandgap energy.[16] The refractive index of the metal-free perovskite DABCO-NH_4Br_3 has a reported bandgap energy of 5.25 eV similar to the 5.12 eV for MDNI and average refractive index from 375 – 650 nm of 1.7.[17] Using this value for the n_{pump}, we estimate a coherence length in MDNI as 0.33 mm, which is comparable to or higher than that of other organic crystals including DAST, DSTMS, OH1, and HMQ-TMS at 800 nm.[15, 18, 19] As we used crystals with thicknesses of approximately 1 mm, this estimate suggests that THz emission from MDNI can be increased using a thinner crystal. Additionally, there is also further opportunity to tune the coherence length of MDNI by pumping at shorter wavelengths where n_{pump} should increase.[18]

5. References

(1) van der Valk, N. C. J.; Wenckebach, T.; Planken, P. C. M. Full Mathematical Description of Electro-Optic Detection in Optically Isotropic Crystals. *Journal of the Optical Society of America B* **2004**, *21* (3), 622. https://doi.org/10.1364/josab.21.000622.

(2) Gallot, G.; Zhang, J.; McGowan, R. W.; Jeon, T. I.; Grischkowsky, D. Measurements of the THz Absorption and Dispersion of ZnTe and Their Relevance to the Electro-Optic Detection of THz Radiation. *Appl Phys Lett* **1999**, *74* (23), 3450. https://doi.org/10.1063/1.124124.

(3) Schleicher, J. M.; Harrel, S. M.; Schmuttenmaer, C. A. Effect of Spin-Polarized Electrons on Terahertz Emission from Photoexcited GaAs. *J Appl Phys* **2009**, *105* (11), 113116. https://doi.org/10.1063/1.3133093.

(4) Yariv, A.; Yeh, P. *Optical Waves in Crystals: Propagation and Control of Laser Radiation*; John Wiley & Sons, Ltd: Hoboken, NJ, 1984.

(5) Xu, G.; Sun, G.; Ding, Y. J.; Zotova, I. B.; Mandal, K. C.; Mertiri, A.; Pabst, G.; Fernelius, N. Investigation of Symmetries of Second-Order Nonlinear Susceptibility Tensor of GaSe Crystals in THz Domain. **2010**. https://doi.org/10.1016/j.optcom.2010.12.025.

(6) Zhang, X.; Jin, Y.; Ma, X. F.; Phys Lett, A. Coherent Measurement of THz Optical Rectification from Electro-Optic Crystals. *Appl. Phys. Lett* **1992**, *61*, 2764–2766. https://doi.org/10.1063/1.108083.

(7) *NIST Chemistry WebBook*. https://webbook.nist.gov/cgi/cbook.cgi?ID=C280579&Mask=80.

(8) Valdivia-Berroeta, G. A.; Tangen, I. C.; Bahr, C. B.; Kenney, K. C.; Jackson, E. W.; Delagange, J.; Michaelis, D. J.; Johnson, J. A. Crystal Growth, Terahertz Generation, and Optical Characterization of EHPSI-4NBS. *J. Phys. Chem. C* **2021**, 125–16097. https://doi.org/10.1021/acs.jpcc.1c01698.

(9) Valdivia-Berroeta, G. A.; Heki, L. K.; Jackson, E. W.; Tangen, I. C.; Bahr, C. B.; Smith, S. J.; Michaelis, D. J.; Johnson, J. A. Terahertz Generation and Optical Characteristics of P-BI. *Opt Lett* **2019**, *44* (17). https://doi.org/10.1364/OL.44.004279.

(10) Valdivia-Berroeta, G. A.; Kenney, K. C.; Jackson, E. W.; Zaccardi, Z.; Tangen, I. C.; Bahr, C. B.; Ho, S. H.; Rader, C.; Smith, S. J.; Michaelis, D. J.; Johnson, J. A. Terahertz Generation of Two Methoxy Stilbazolium Crystals: MBST and MBSC. *Opt Mater (Amst)* **2021**, *117*, 111119. https://doi.org/10.1016/j.optmat.2021.111119.

(11) Zaccardi, Z. B.; Tangen, I. C.; Valdivia-Berroeta, G. A.; Bahr, C. B.; Kenney, K. C.; Rader, C.; Lutz, M. J.; Hunter, B. P.; Michaelis, D. J.; Johnson, J. A. Enabling High-Power, Broadband THz Generation with 800-Nm Pump Wavelength. *Opt Express* **2021**, *29* (23), 38084. https://doi.org/10.1364/oe.437421.

(12) Ofori-Okai, B. K.; Sivarajah, P.; Ronny Huang, W.; Nelson, K. A. THz Generation Using a Reflective Stair-Step Echelon. *Opt Express* **2016**, *24* (5), 5057. https://doi.org/10.1364/oe.24.005057.

(13) Zhang, B.; Chai, S.; Fang, Z.; Xia, C.; Kong, D.; Wang, J.; Liu, H.; Zhu, C.; Wang, X.; Ruan, C.; Hafez, H. A.; Chai, X.; Ibrahim, A.; Mondal, S.; Férachou, D.; Ropagnol, X.; Schubert, O.; Hohenleutner, M.; Langer, F.; Urbanek, B.; Lange, C.; Huttner, U.; Golde, D.; Meier, T.; Kira, M.; Koch, S. W.; Huber, R.; Huang, R.; Fallahi, A.; Wu, X.; Cankaya, H.; Calendron, A.; Ravi, K.; Zhang, D.; Nanni, E. A.; Hong, K.; Kaertner, F. X.; Matsunaga, R.; Tsuji, N.; Fujita, H.; Sugioka, A.; Makise, K.; Uzawa, Y.; Terai, H.; Wang, Z.; Aoki, H. Highly Efficient Generation of 0.2 MJ Terahertz Pulses in Lithium Niobate at Room Temperature with Sub-50 Fs Chirped Ti:Sapphire Laser Pulses. *Optics Express, Vol. 26, Issue 6, pp. 7107-7116* **2018**, *26* (6), 7107–7116. https://doi.org/10.1364/OE.26.007107.

(14) Ponseca, C. S.; Arlauskas, A.; Yu, H.; Wang, F.; Nevinskas, I.; Du Da, E.; Vaičaitis, V.; Eriksson, J.; Bergqvist, J.; Liu, X. K.; Kemerink, M.; Krotkus, A. N.; Inganas, O.; Gao, F. Pulsed Terahertz Emission from Solution-Processed Lead Iodide Perovskite Films. *ACS Photonics* **2019**, *6* (5), 1175–1181. https://doi.org/10.1021/acsphotonics.8b01693.

(15) Jazbinsek, M.; Puc, U.; Abina, A.; Zidansek, A., Organic Crystals for THz Photonics. *Applied Sciences-Basel* **2019,** *9* (5), 882.

(16) Singh, J. K.; Mandal, S. K.; Banerjee, G., Refractive index of different perovskite materials. *Journal of Materials Research* **2021,** *36* (9), 1773-1793.

(17) Cui, Q. Y.; Song, X.; Liu, Y. C.; Xu, Z.; Ye, H. C.; Yang, Z.; Zhao, K.; Liu, S. Z., Halide-modulated self-assembly of metal-free perovskite single crystals for bio-friendly X-ray detection. *Matter* **2021,** *4* (7), 2490-2507.

(18) Kim, D.; Kim, W. T.; Han, J. H.; Lee, J. A.; Lee, S. H.; Kang, B. J.; Jazbinsek, M.; Yoon, W.; Yun, H. S.; Kim, D.; van Bezouw, S.; Campo, J.; Wenseleers, W.; Rotermund, F.; Kwon, O. P., Wide-Bandgap Organic Crystals: Enhanced Optical-to-Terahertz Nonlinear Frequency Conversion at Near-Infrared Pumping. *Advanced Optical Materials* **2020,** *8* (10), 1902099.

(19) Kim, S. J.; Kang, B. J.; Puc, U.; Kim, W. T.; Jazbinsek, M.; Rotermund, F.; Kwon, O. P., Highly Nonlinear Optical Organic Crystals for Efficient Terahertz Wave Generation, Detection, and Applications. *Advanced Optical Materials* **2021,** *9* (23), 2101019.
